# Tractography of Porcine Meniscus Microstructure Using High-Resolution Diffusion Magnetic Resonance Imaging

**DOI:** 10.3389/fendo.2022.876784

**Published:** 2022-05-10

**Authors:** Jikai Shen, Qi Zhao, Yi Qi, Gary Cofer, G. Allan Johnson, Nian Wang

**Affiliations:** ^1^Department of Biomedical Engineering, Duke University, Durham, NC, United States; ^2^School of Life Sciences, Westlake University, Hangzhou, China; ^3^Physical Education Institute, Jimei University, Xiamen, China; ^4^Department of Radiology, Duke University School of Medicine, Durham, NC, United States; ^5^Department of Radiology and Imaging Sciences, Indiana University School of Medicine, Indianapolis, IN, United States; ^6^Stark Neurosciences Research Institute, Indiana University, Indianapolis, IN, United States

**Keywords:** DTI, knee, tractography, meniscus, connectivity, segmentation

## Abstract

To noninvasively evaluate the three-dimensional collagen fiber architecture of porcine meniscus using diffusion MRI, meniscal specimens were scanned using a 3D diffusion-weighted spin-echo pulse sequence at 7.0 T. The collagen fiber alignment was revealed in each voxel and the complex 3D collagen network was visualized for the entire meniscus using tractography. The proposed automatic segmentation methods divided the whole meniscus to different zones (Red-Red, Red-White, and White-White) and different parts (anterior, body, and posterior). The diffusion tensor imaging (DTI) metrics were quantified based on the segmentation results. The heatmap was generated to investigate the connections among different regions of meniscus. Strong zonal-dependent diffusion properties were demonstrated by DTI metrics. The fractional anisotropy (FA) value increased from 0.13 (White-White zone) to 0.26 (Red-Red zone) and the radial diffusivity (RD) value changed from 1.0 × 10^-3^ mm^2^/s (White-White zone) to 0.7 × 10^-3^ mm^2^/s (Red-Red zone). Coexistence of both radial and circumferential collagen fibers in the meniscus was evident by diffusion tractography. Weak connections were found between White-White zone and Red-Red zone in each part of the meniscus. The anterior part and posterior part were less connected, while the body part showed high connections to both anterior part and posterior part. The tractography based on diffusion MRI may provide a complementary method to study the integrity of meniscus and nondestructively visualize the 3D collagen fiber architecture.

## Introduction

Magnetic resonance imaging (MRI) is the modality of choice for diagnosing meniscal tears with both high sensitivity and specificity (1, 2). Conventional morphological MRI relies on the assessment of surface integrity and sub-surface signal intensity as indicators of tissue defects, which is qualitative and has limitations to detect the meniscal composition changes before surface breakdown or small tears (3). Relaxation time-based quantitative MRI such as T1 and T2 mappings have been widely used to investigate the compositional tissue features beyond morphology and structure (4, 5).

Diffusion MRI (dMRI) has been extensively used to reveal the microstructure of different tissues due to its sensitivity to the microscopic cellular organization (6). Diffusion based tractography has been performed to identify anatomic connections in mouse brains (7). The derived structural connectivity maps provide insight into the network of interconnected brain regions and ultimately lead to improved diagnosis of various brain disorders (8). Recently, dMRI and tractography in musculoskeletal system has attracted more and more attention to investigate the tissue microstructure, local collagen fiber alignment, and the 3D collagen network (9–11). To the best of our knowledge, noninvasively probing the 3D collagen fiber architecture and connections among different parts of the porcine meniscus have not been reported yet, probably due to a few reasons. First, the tractography is challenging in menisci due to the low fractional anisotropy (FA) and short T2 (4). Second, the spatial resolution is often limited for meniscus MRI due to its thin-layer anatomical structure. Third, the structural connectivity analysis relies on segmenting the meniscus to different sub-regions (12). However, structural connection by diffusion tensor imaging (DTI) reveals not only the local tissue properties but also the 3D fiber network through the whole tissue area.

Besides tractography, the scalar metrics from DTI model are known to be related to the tissue microstructure (8, 13, 14). For instance, the FA and mean diffusivity (MD) are found to be sensitive to the collagen architecture and glycosaminoglycan (GAG) content in different zones of cartilage (15). Exploring the FA and MD variations at different zones of meniscus may help to better understand the microstructure of meniscus, which requires to segment the tissue manually or automatically. Cooper’s classification is one of the most commonly used meniscal classification systems based on the blood supply (16). According to the classification, menisci can be divided into Red-Red zone (outer third of the meniscus), Red-White zone (middle third of the meniscus), and White-White zone (inner third of the meniscus) (17).

Menisci often show low signal intensity on MR images due to the short T2 relaxation time and conventional MRI cannot adequately distinguish red zone and white zone because of little contrast difference between these zones (18). It is possible to visualize enhancement selectively in the red zone of the meniscus using ultrashort echo time (UTE) pulse sequence, but dividing the meniscus to white and red zones is still challenging (19). Conventionally, the meniscus can be manually segmented to different zones, but it is an expertise-intensive and time-consuming process (20). Numerous subjective interpretations for separating adjacent structures with similar image contrasts result in low repeatability and less efficiency. Therefore, automation of the segmentation process is highly desirable.

In this study, we first acquired 3D diffusion-weighed spin-echo pulse sequence to probe the microstructure of porcine meniscus. We then developed a segmentation method with rotational and radial directions to divide the meniscus into different sub-regions. The automatic segmentation method was further validated in the human knee MRI images obtained from the Osteoarthritis Initiative (OAI) database. The water diffusion properties derived from DTI have been quantified at different zones of meniscus. Diffusion tractography was performed through whole meniscus to visualize the 3D collagen fiber network. Combining tractography and automatic segmentation, we were able to observe the structural connections among different areas of the meniscus.

## Materials and Methods

### Specimen Preparation

Five normal porcine menisci were harvested shortly after the sacrifice of mature porcine knee joints obtained from a local abattoir. The specimens were then immersed in a phosphate buffered solution (PBS) solution of 0.5% gadoteridol (Prohance® Bracco Diagnostics Inc., Princeton, NJ) to shorten the T1 relaxation time to about 110 ms and to reduce the scan time (21).

### Microscopic MRI (µMRI) Protocols

The specimens were scanned on a 7.0 T small animal MRI system (Magnex Scientific, Yarnton, Oxford, UK) equipped with 650 mT/m Resonance Research gradient coils (Resonance Research Inc., MA, USA). RF transmission and reception were achieved using a homemade solenoid coil (10 × 5 × 5 cm^3^) (7). A modified 3D Stejskal-Tanner diffusion-weighted spin-echo pulse sequence to support k-space under sampling was performed for diffusion MRI scans (22). The imaging parameters were: TR = 100 ms, Matrix size = 512 × 256 × 256, FOV = 64 ×32 × 32 mm^3^, TE = 13.0 ms, 125 μm isotropic spatial resolution, b value = 1000 s/mm^2^ with 81 diffusion gradient encoding directions and 8 non-diffusion-weighted (b0) measurements. The gradient separation time was 5.5 ms and the diffusion gradient duration time was 4.5 ms for all scans. Acceleration factor (AF) of 8.0 was used for a sparsity approach, which has been described in detail previously (22). The scan time was 20.1 hours. The maximum gradient amplitude was about 60 G/cm. The diffusion gradient orientations (distributed over half sphere) were optimized to ensure the uniformity of encoding directions on the shell. The representative diffusion-weighted images (DWIs) and the signal intensity variations at different gradient orientations were shown in **Supplementary Figure 1**. The temperature was monitored throughout all the scans and the fluctuation was less than 1°C. T2-weighed images were extracted from the non-diffusion-weighted (b0) images for segmentation purpose only.

3D multi-echo gradient echo (MGRE) scans were acquired at the spatial resolution of 250 μm with matrix size = 96 × 60 × 40 and 24 echoes (TE = 1.80/1.43/34.69 ms); FOV = 48 mm × 30 mm × 20 mm, flip angle = 30°, bandwidth (BW) = 125 kHz, and TR = 100 ms. The scan time was about 16 minutes. The T2*-weighted image derived from MGRE scans were used for segmentation purpose only.

### Human Knee MRI

To validate the robustness of the automatic segmentation method, the human knee MRI images were obtained from the OAI database, which is available for public access (https://nda.nih.gov/oai/). A sagittal 3D WE DESS (water excitation double-echo steady-state) MR dataset of the knee featuring a high spatial resolution (0.37 x 0.37 mm^2^ in plane, 0.7 mm slice thickness) was selected. The meniscus mask was manually drawn in ITK-SNAP software.

### Manual Segmentation

Manual segmentation is common for meniscus analysis. However, due to the irregular shape of the meniscus, the accuracy of manual segmentation has not been investigated in detail. The porcine meniscus was manually segmented using ITK-SNAP software based on DWI. The meniscus was divided slice by slice into three zones in the Cartesian acquisition coordinate frame according to Cooper’s classification: Red-Red (R-R) zone, Red-White (R-W) zone, White-White (W-W) zone (**Supplementary Figure 1**).

### Automatic Segmentation

#### Radial Segmentation

**Figure 1:**
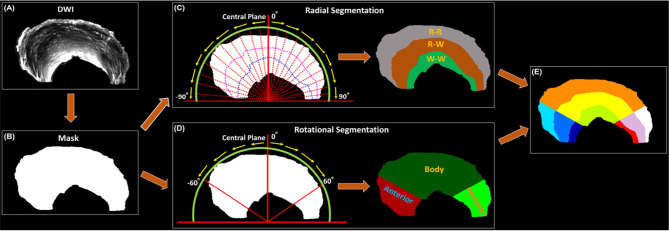
The automatic segmentation process used in this study, from the acquired DWI **(A)** to the 9 different areas of meniscus **(E)**. Both Radial Segmentation **(C)** and Rotational Segmentation **(D)** were derived from the binary mask **(B)**. These two methods were further combined to divide the whole meniscus to 9 regions **(E)**. R-R, Red-Red zone; R-W, Red-White zone; W-W, White-White zone.

The meniscus was divided into three zones according to Cooper’s classification: Red-Red (R-R) zone, Red-White (R-W) zone, White-White (W-W) zone. First, a binary mask (**Figure 1B**) was generated based on the DWI image (**Figure 1A**). The whole meniscus mask was rotated clockwise (0° - 90°) and anticlockwise (-90° - 0°) with the step size of 0.5° with respect to the central plane (**Figure 1C**). A 2D cross-section image by the central plane was generated by each rotation. Each 2D image was then trisected to R-R, R-W, and W-W zones evenly based on the length in the radial direction (red dash lines in **Figure 1C** and **Supplementary Figure 2E**). Last, all the 2D segmented images were reassembled into the final 3D zonal segmentation. The automatic segmentation was implemented in Matlab (MathWorks, Natick, MA). Compared to automatic segmentation, the R-W and R-R zones were partially covered by W-W zone using manual segmentation (**Supplementary Figure 2**).

#### Rotational Segmentation

The whole meniscus could also be divided into 3 parts: anterior part, body part, and posterior part. To achieve this, the similar rotations were performed clockwise and anticlockwise for a certain degree to distinguish the three different parts (**Figure 1D**). Then the anterior, body, and posterior parts were segmented based on the rotation angle (60° in the current study). Note that no specific rotation degree can be defined as the ground truth, this angle was set to be adjustable for the Rotational Segmentation method (**Supplementary Figure 3**). In addition, we combined the Radial Segmentation and Rotational Segmentation methods to further divide meniscus to 9 regions, where each zone (R-R, R-W, or W-W) contained anterior, body, and posterior parts (**Figure 1E**).

### Diffusion Metrics of Meniscus

All the DWIs were registered to the baseline images (b0). The DTI model was used to capture the primary diffusion direction of the collagen fiber. The scalar indices including FA, MD, axial diffusivity (AD), and radial diffusivity (RD) were calculated for both manual and automatic segmentation. Deterministic fiber tracking was performed for the whole meniscus as well as the ROIs at three different zones (manually drawn in ITK-SNAP software). The propagation process was repeated until the tracking trajectory exceeded the turning angle greater than 45°. The connection strength map among different zones and parts was generated after whole meniscus tractography. All fiber tracking operations were performed using Diffusion Spectrum Imaging (DSI) studio toolbox (https://dsi-studio.labsolver.org/) (23).

### Statistics

The volume and DTI metrics from 5 menisci were reported with their mean value and standard deviation. Direct comparison of volume and DTI metrics among different zones (R-R, R-W, and W-W) were performed using one-way ANOVA analysis in MATLAB, where *p*-value below 0.05 stands for a significant difference of DTI metrics among different zones.

## Results

**Figure 2:**
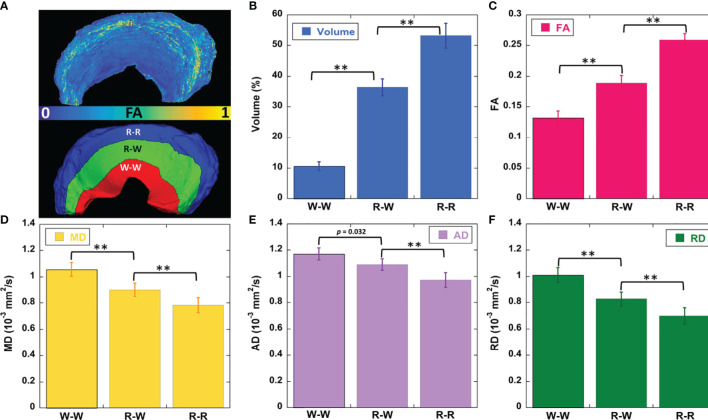
The FA and segmentation 3D rendering images **(A)**, volume **(B)**, DTI metrics of FA **(C)**, mean diffusivity **(D)**, axial diffusivity **(E)**, and radial diffusivity **(F)** values at different zones. FA value gradually decreases from R-R to W-W zone **(C)**. The diffusivity metrics exhibit the opposite trends **(D–F)**. ** stands for *p*-value <0.001. R-R, Red-Red zone; R-W, Red-White zone; W-W, White-White zone.

**Figure 2** showed the mean volumes and quantitative DTI metrics (FA, MD, AD, and RD) in three different zones. The heterogeneous appearance of the meniscus at different zones was evident in FA image (2A). The volume (2B) gradually increased from W-W zone (10.4%) to R-R zone (53.1%). Similar to the volume, the FA values (2C) gradually increased from W-W zone (0.13) to R-R zone (0.26) with a 100% increase. In contrast, MD, AD, and RD values (2D-2F) gradually decreased from W-W zone to R-R zone. For instance, the RD values changed from 1.0 × 10^-3^ mm^2^/s (W-W zone) to 0.7 × 10^-3^ mm^2^/s (R-R zone), which decreased 30%. Significant differences (p < 0.01) of volume and all the DTI metrics had been found among three different zones. The values of volumes and DTI metrics were summarized in **Table 1**.

**Table 1:** The volumes and DTI metrics at different zones of meniscus.

Zones	Volume (%)	FA	MD (10^-3^ mm^2^/s)	AD (10^-3^ mm^2^/s)	RD (10^-3^ mm^2^/s)
W-W	10.40 ± 1.41	0.13 ± 0.01	1.06 ± 0.09	1.17 ± 0.06	1.00 ± 0.06
R-W	36.50 ± 2.80	0.19 ± 0.02	0.90 ± 0.07	1.09 ± 0.06	0.83 ± 0.05
W-W	53.10 ± 4.08	0.26 ± 0.02	0.78 ± 0.06	0.97 ± 0.07	0.70 ± 0.05

**Figure 3:**
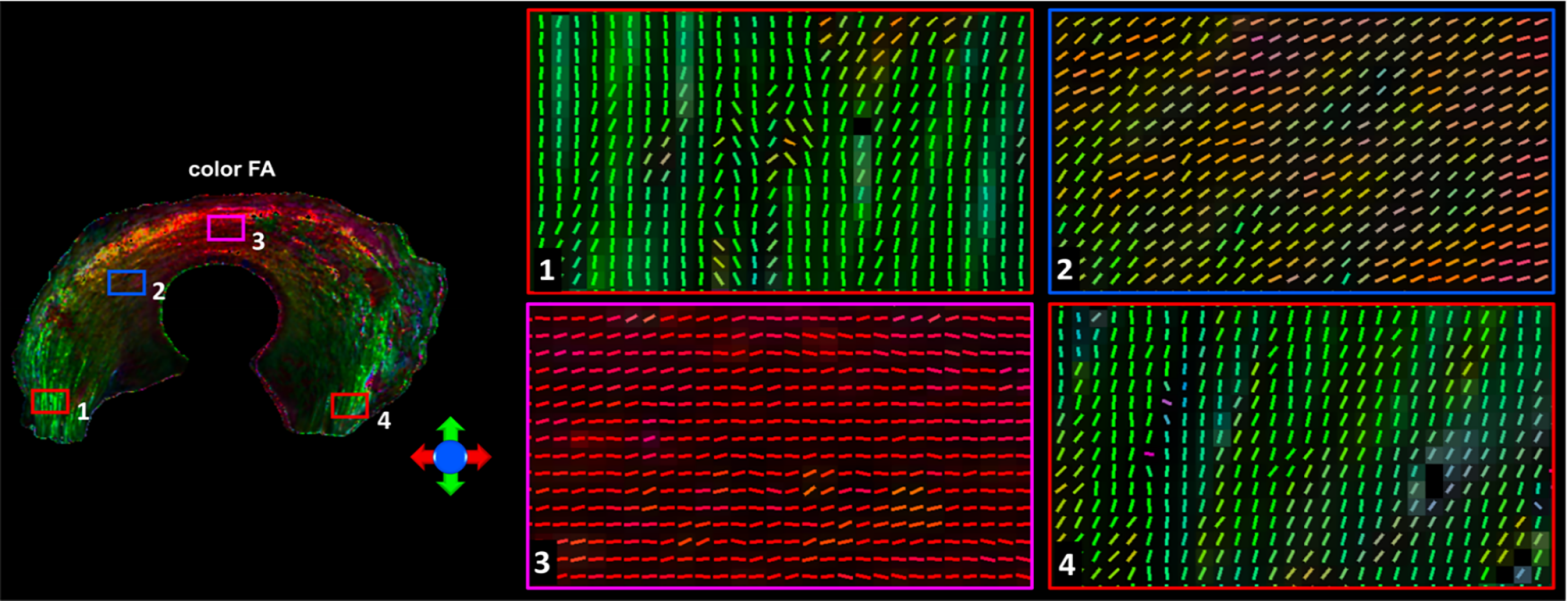
The color-FA and the fiber orientation images of meniscus at different regions. Red for horizontal fiber, green for vertical, blue for inside-out. The collagen fiber orientations were shown at 4 different regions.

**Figure 4:**
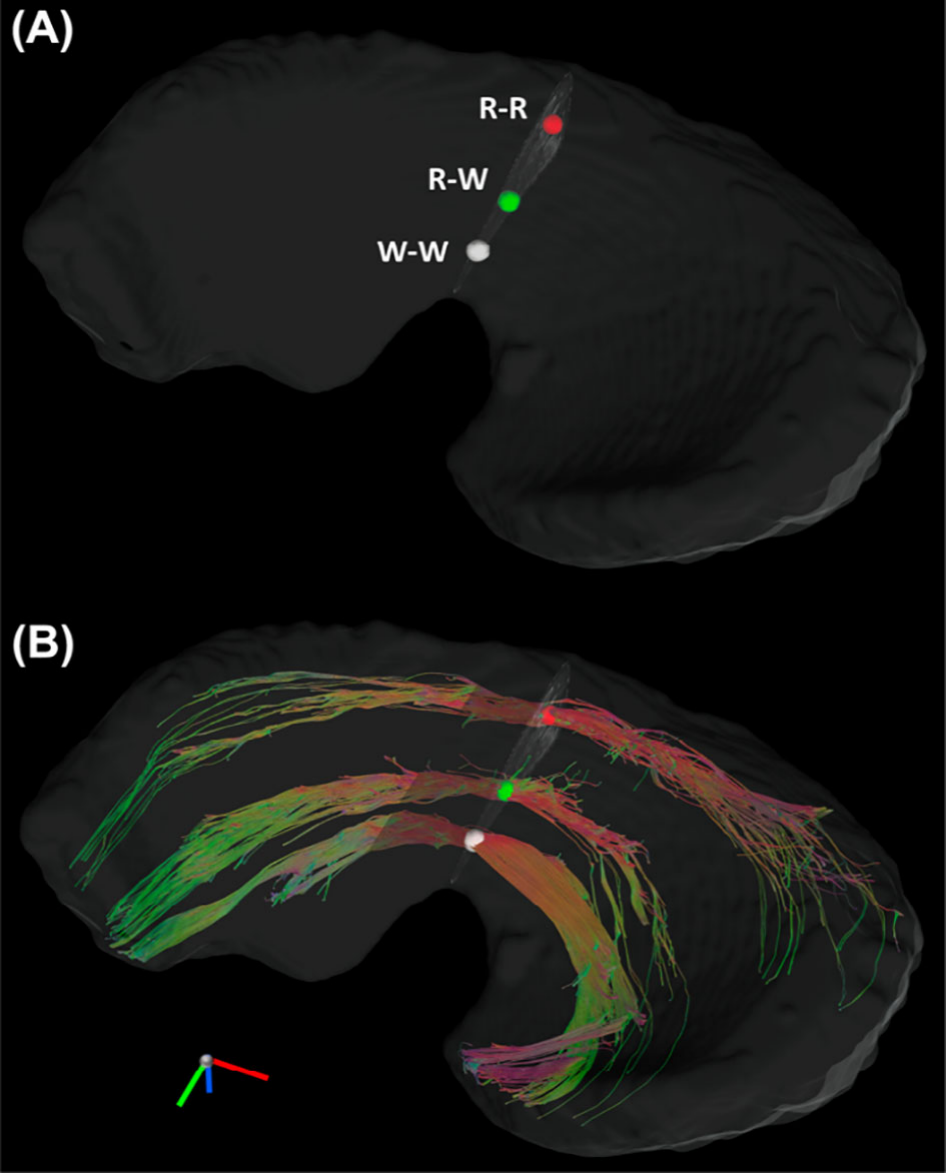
The Tracts **(B)** from three seeding regions **(A)**: red area in R-R zone, white area in R-W zone, and white area from W-W zone. R-R, Red-Red zone; R-W, Red-White zone; W-W, White-White zone.

**Figure 3** showed the color-FA and the fiber orientation images of meniscus in different regions. The collagen fiber exhibited orthotropic directions between the anterior part (ROI 1, green color) and the body part (ROI 3, red color), between the posterior part (ROI 4, green color) and the body part. The fiber directions were found to gradually change from anterior part to the body part (ROI 2). These fiber directions were also evident from the individual tracts (**Figure 4**), where the seeding regions (**Figure 4A**) are from R-R zone (red area), R-W zone (green area), and W-W zone (white area), respectively. The circumferential collagen fibers were found in all three zones.

**Figure 5:**
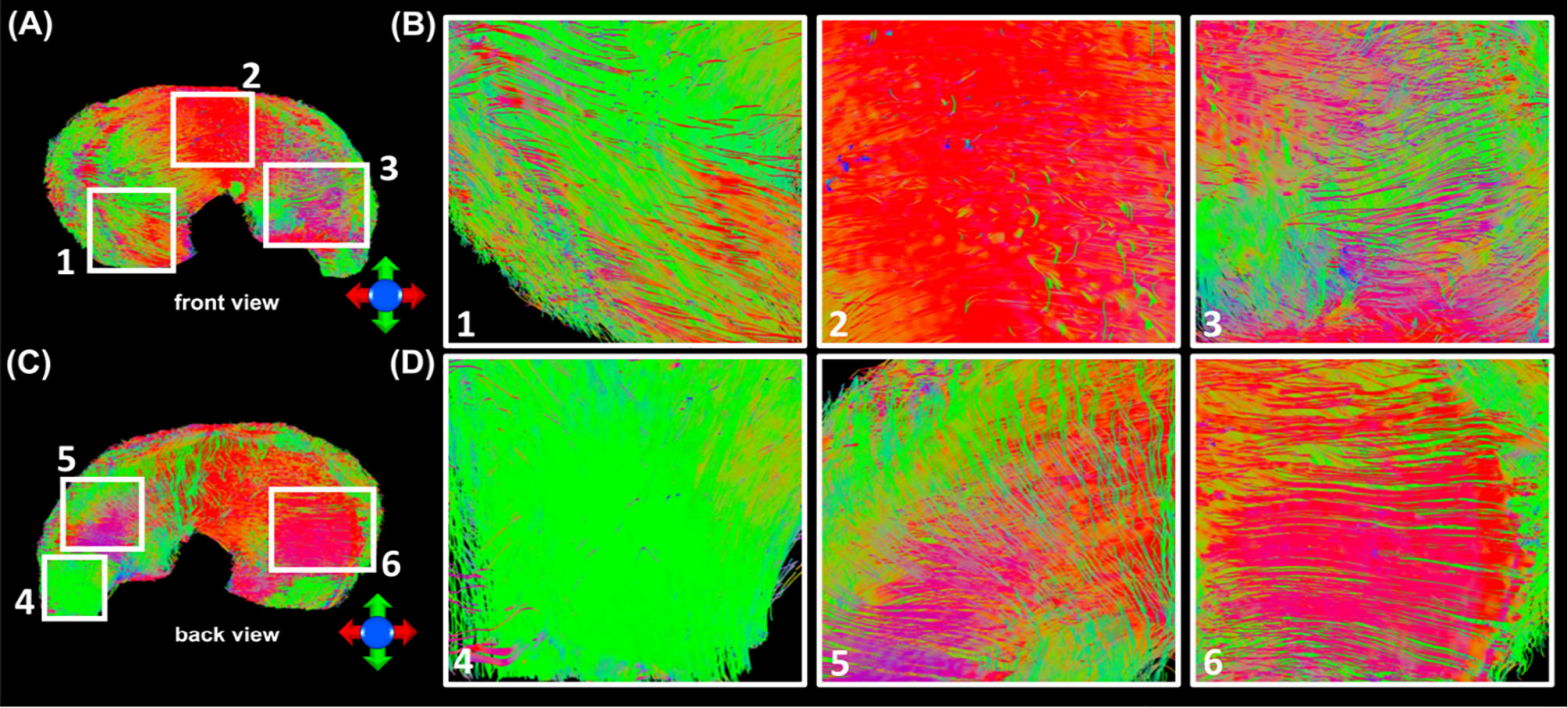
The entire meniscus 3D collagen fiber network **(A, C)** resolved by tractography. The coexistence of both radial and circumferential collagen fibers was found from ROIs 1 and 3 (front view, **B**), and ROIs 5 and 6 (back view, **D**).

In order to explore the entire meniscus 3D collagen fiber network, the tractography were performed in the whole meniscus area (**Figures 5A**, **C**, front and back view). The tractography showed similar collagen fiber architectures as **Figure 4**: the circumferential collagen fibers through anterior part to posterior part. However, the whole meniscus tracts revealed the coexistence of both radial and circumferential collagen fibers, especially in ROIs of 1 and 3 (front view), 5 and 6 (back view). The consistent tractography and fiber orientation results were demonstrated in all five menisci (**Supplementary Figure 4**).

**Figure 6:**
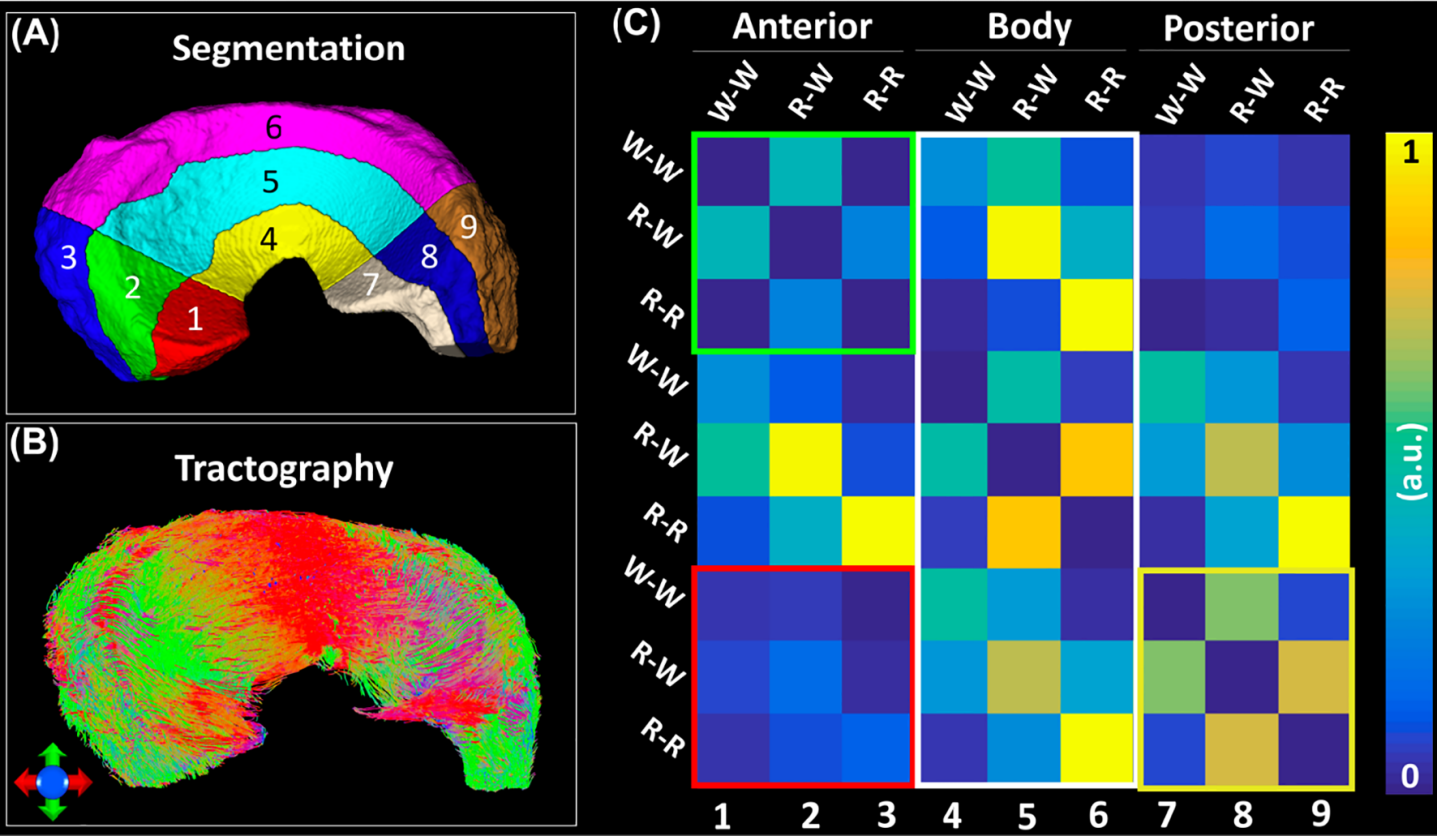
The structural connection heatmap of meniscus **(C)** obtained by the automatic parcellation **(A)** and tractography **(B)**.

**Figure 6** exhibited the automatic segmentation (6A), the diffusion tractography (6B), and the connection heatmap of meniscus (6E). Several distinct characteristics can be identified from the tractography images and heatmap. First, the anterior part showed low connections to the posterior part (red box in 6C), while the body part showed high connections to both anterior part and posterior part (white box in 6C). Second, in the same part (anterior, body, or posterior), the R-W zone exhibited high connections to the adjacent parts (green box and yellow box).

In order to explore the robustness of the automatic segmentation method, the Radial Segmentation was also applied to T2- and T2*-weighted images (**Supplementary Figure 5**). We further extended our methods for human knee meniscus segmentation (**Supplementary Figure 6**). Both Radial Segmentation and Rotational Segmentation (**Supplementary Figures 6B, C**) methods showed visually comparable results to porcine meniscal segmentations.

## Discussion

MRI has emerged as an invaluable component of pathogenesis research in meniscal tear. Quantitative MRI (qMRI) has been applied to study the correlation between the parametric mapping and the severity of tissue degradation (4, 5). Most of the qMRI used to access the biochemical status of the menisci are based on relaxation times, such as T2, T2*, T1rho, and T1, which are challenging to estimate the local collagen fiber alignment directly (24, 25). In order to investigate the comprehensive collagenous fibril texture of meniscus, other imaging modalities such as polarized light imaging (PLM), reflectance confocal microscopy, and scanning electron microscopy (SEM) have been used in previous studies (26–29). While it is well appreciated that complex fiber structures (circumferential and radial) exist within the meniscus using these technologies, they are often limited in a small region and presented as two-dimensional (2D) images. Recently, DTI has been used to study the tissue microstructure and quantify the local collagen fiber direction in knee joint (21, 30–32). The local fiber orientation from DTI model affords an alternative to probe the complex collagen fiber directions of the whole meniscus.

Tractography, as a promising technology to visualize the complex 3D fiber network, has been recently applied to individual connective tissues in knee joint, such as cartilage, anterior cruciate ligament (ACL), and tendon (9, 10, 33, 34). However, the application of tractography to probe the meniscal microstructure is rare, probably due to the low signal-to-noise ratio (SNR) and short T2* values (35–37). To overcome this issue, the menisci were images in a preclinical 7T system with powerful gradients to increase the SNR by minimizing the TE value to 13.0 ms. With the proposed automatic segmentation methods, the connection heatmap has been generated to quantify the connections among different zones (R-R, R-W, and W-W) and different parts (anterior, body, and posterior). Compared to the exisiting qMRI methods focusing on the local change of the tissue properties, the alteration of the connections in meniscus may provide a complementary method to study the integrity of meniscus and meniscal tears in future studies.

It has been reported that FA is sensitive to collagen architecture and MD is sensitive to GAG content in cartilage (31). Cartilage has been found to show depth-dependent response to the degradation, especially in the superficial zone at early osteoarthritis (OA) (18). The variations of DTI metrics at different parts and zones may be related to the meniscal microstructure and composition changes after degradation. Several automatic and semi-automatic segmentation methods have been developed to segment the cartilage, bone, and meniscus in knee joint (38, 39). Although these methods provide excellent segmentation accuracy to distinguish meniscus to other soft tissues, automatically dividing the meniscus to different zones and parts is still limited. Unlike the articular cartilage, which can be divided into three zones according to the collagen fiber directions, both red zone and white zone show similar fiber directions in meniscus (40). The lack of apparent image contrasts between red zone and white zone makes the segmentation more challenging.

In this study, we adapted Cooper’s classification for the segmentation. The segmentation allows us to investigate the diffusion properties of menisci at different zones or parts. It also helps us to obtain the heatmap to show the structural connections among different regions. The proposed segmentation, even is not a gold standard, does afford a convenient way for quantitative analysis of meniscus diffusion properties. The proposed segmentation method requires a simple binary mask of the meniscus, which is relatively easy to obtain from MRI scans, such as T2-weighted image, T2*-weighted image, and DWI (41). The automatic segmentation method may not be limited to MRI and can be applied to different imaging modalities as long as the binary mask is available. Furthermore, the method also shows robust results for human meniscus segmentation, which suggests that our method holds the potential to segment meniscus to different sub-regions for human studies (42).

There are a few limitations in our study. First, the sample size is small due to the extremely long scan time, the consistent collagen fiber alignment and tractography were demonstrated in all five specimens. Second, the dMRI was acquired with a preclinical setting that may not relevant to the clinical study. To the best of our knowledge, it’s still challenging to achieve high quality meniscus dMRI and tractography for *in vivo* human studies. Advanced acquisition technologies, stronger gradients, higher magnetic field, and novel reconstruction methods shed the light to bridge the gap (43). Third, although the collagen fiber architecture revealed by dMRI is consistent with other imaging modalities in previous studies, complete validation of MRI findings using other imaging modalities is warranted in future studies. Last, this Cooper’s classification is originally defined for human meniscus, our method may be improved for animal meniscal studies when further shape information about different species is known.

In conclusion, the porcine meniscus microstructure was investigated using a 3D diffusion-weighed spin-echo pulse sequence. Strong zonal-dependent diffusion properties were demonstrated by DTI metrics (FA, MD, AD, and RD). The complex 3D collagen fiber architecture of the entire meniscus was visualized by diffusion tractography. Combining tractography and automatic segmentation method, we were able to observe the structural connections among different areas of the meniscus. It may offer a novel method to evaluate the local meniscus tears and address the alteration of connections among different regions of the meniscus.

## Data Availability Statement

The raw data supporting the conclusions of this article will be made available by the authors, without undue reservation.

## Ethics Statement

The animal study was reviewed and approved by institutional animal care and use committee (IACUC).

## Author Contributions

JS: Methodology, Formal analysis, Writing-review and editing. QZ: Editing, Formal analysis, Validation. YQ: Specimen preparation, Methodology, Writing-review and editing. GC: MRI acquisition, Writing-review and editing. GJ: Conceptualization, Investigation, Writing-review and editing, Supervision, Funding acquisition. NW: Conceptualization, Methodology, MRI acquisition, Formal analysis, Investigation, Writing -original draft, Funding acquisition, Writing-review and editing. All authors contributed to the article and approved the submitted version.

## Funding

This work was supported by the NIH/NIBIB National Biomedical Technology Resource Center P41 EB015897 (to GJ), Charles E. Putman MD Vision Award of the Department of Radiology, Duke University School of Medicine (to NW and Charles E. SPritzer), and Strategic Research Initiative (SRI) IUH and Indiana University School of Medicine (to NW).

## Conflict of Interest

The authors declare that the research was conducted in the absence of any commercial or financial relationships that could be construed as a potential conflict of interest.

## Publisher’s Note

All claims expressed in this article are solely those of the authors and do not necessarily represent those of their affiliated organizations, or those of the publisher, the editors and the reviewers. Any product that may be evaluated in this article, or claim that may be made by its manufacturer, is not guaranteed or endorsed by the publisher.

## Abbreviations

DTI, diffusion tensor imaging; FA, fractional anisotropy; MD, mean diffusivity; AD, axial diffusivity; RD, radial diffusivity; GAG, glycosaminoglycan; DSI, Diffusion spectrum imaging; UTE, ultra-short echo; R-R, Red-Red; R-W, Red-White; W-W, White-White.
