# Supplementary material for: Tractography of Porcine Meniscus Microstructure Using High-Resolution Diffusion Magnetic Resonance Imaging
Source: Front Endocrinol (Lausanne). 2022 May 10;13:876784. doi: 10.3389/fendo.2022.876784 (PMC9127075; doi:10.3389/fendo.2022.876784)
Supplement: Supplementary file 1 [file DataSheet_1.docx]

**Supplemental Information**

**
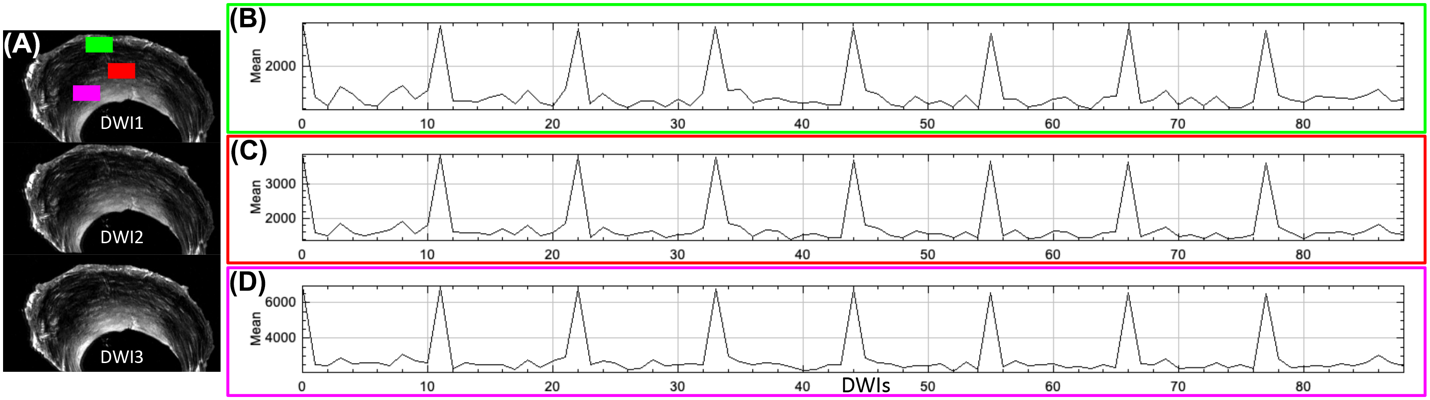
**

**Supporting Information Figure S1.** The representative DWIs and the signal intensity variations at different gradient orientations.


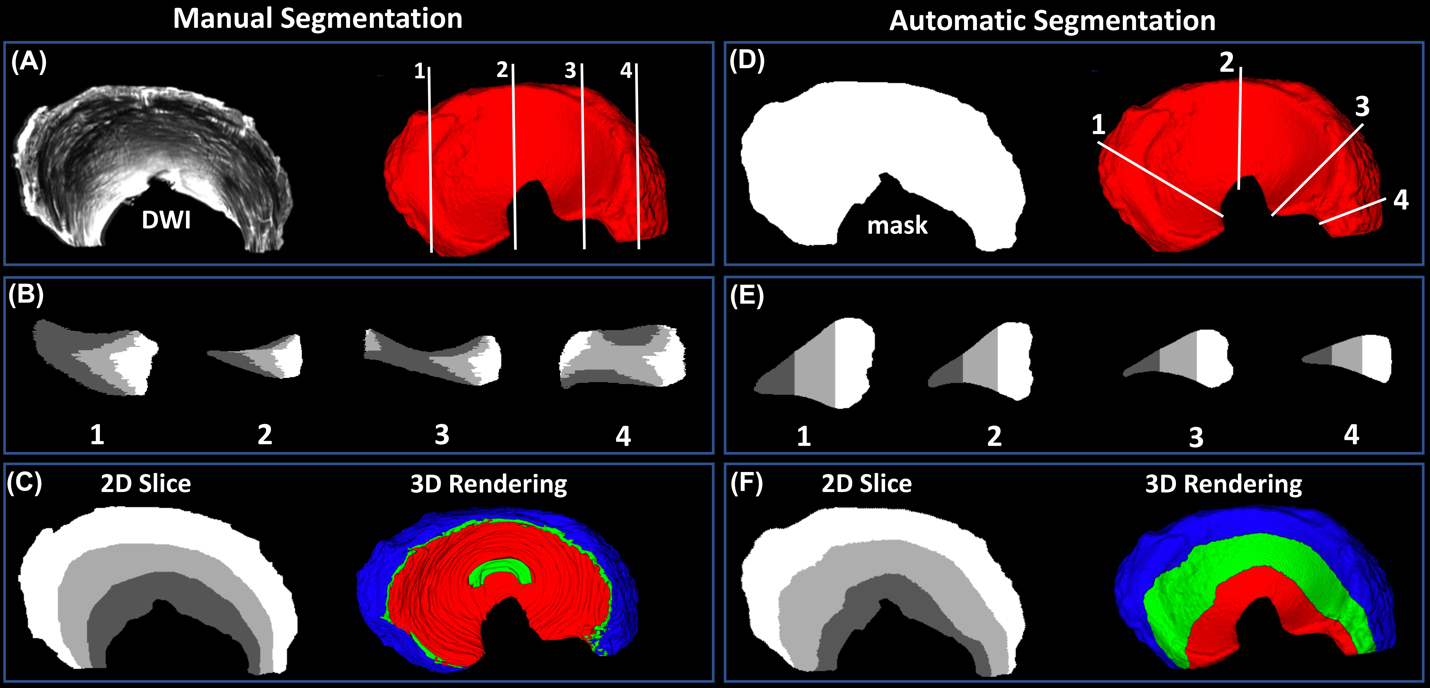


**Supporting Information Figure S2.** The flow diagrams of manual segmentation (A-C) and automatic segmentation (D-F). Both 2D slices and 3D rendering of meniscus were shown for visualization.


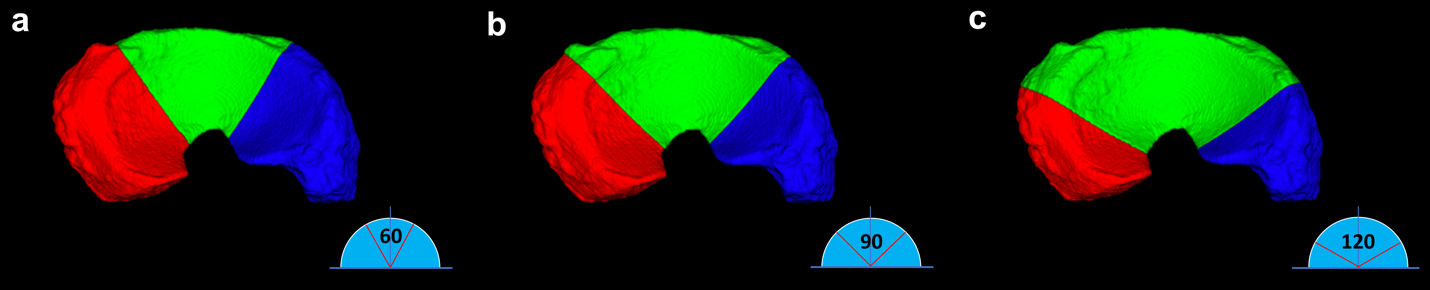


**Supporting Information Figure S3.** The angle for Rational Segmentation is adjustable.


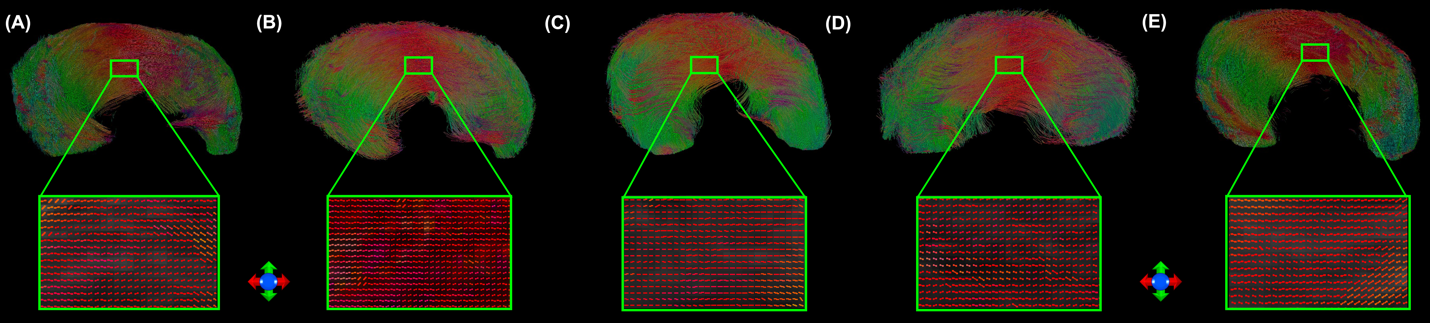


**Supporting Information Figure S4.** The consistent tractography and fiber orientation results in all five menisci.


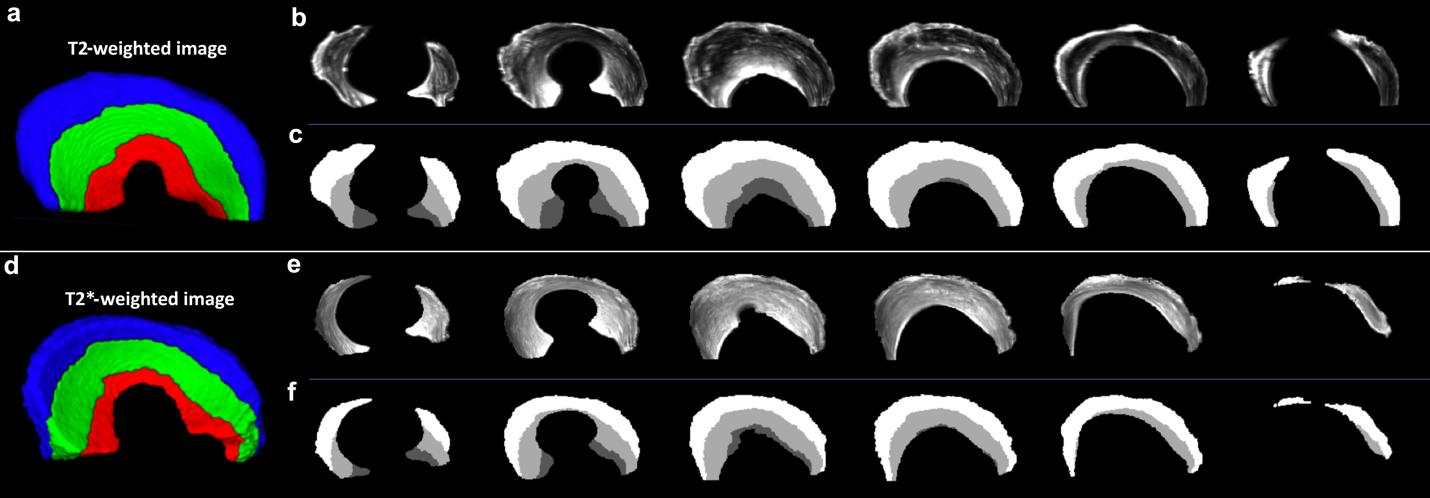


**Supporting Information Figure S5.** The Radial Segmentation is extended to T2- and T2*-weighted images.


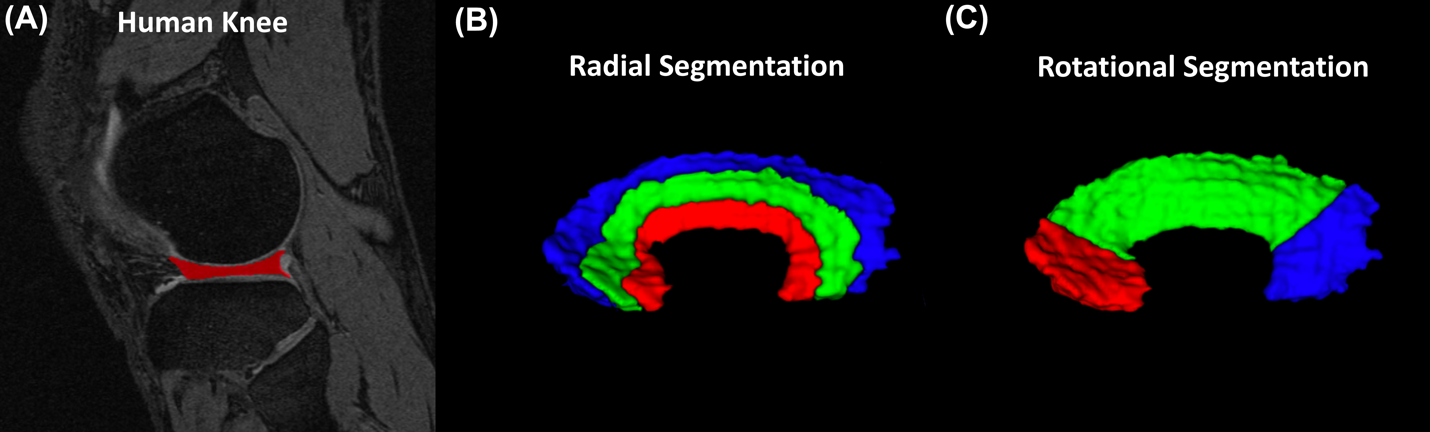


**Supporting Information Figure S6.** The proposed methods used in human knee meniscus segmentation. The mask (A) was manually drawn and then used for Radial segmentations (B) and Rotational Segmentation (C).
